# Partial regression of large anterior scleral staphyloma secondary to rhinosporidiosis after corneoscleral graft - a case report

**DOI:** 10.1186/s12886-018-0725-2

**Published:** 2018-02-27

**Authors:** Elaine Fiod Costa, Luciano Moreira Pinto, Marcos Adriano Garcia Campos, Thais Mota Gomes, Gyl Eanes Barros Silva

**Affiliations:** 1 Departamento de Medicina I, Universidade Federal do Maranhão, Praça Gonçalves Dias, 21 – Centro, São Luis,, MA 65020-240 Brazil; 2 Departamento de Oftalmologia, Universidade Federal de São Paulo, Rua Botucatu, 821 – Vila Clementino, São Paulo, SP 04023-062 Brazil

**Keywords:** Rhinosporidiosis, Staphyloma, Corneal grafting, Case report

## Abstract

**Background:**

Rhinosporidiosis is a rare chronic infection of the mucous membranes caused by the *Rhinosporidium seeberi*. Approximately 15% of cases of rhinosporidiosis are ocular, occurring mainly in the tarsal conjunctiva. There are only 11 cases of scleral melt with staphyloma formation associated with bulbar conjuctival oculosporidiosis and none of them was associated with partial regression of the scleral ectasia after a corneoscleral tectonic graft.

**Case presentation:**

a 13-year-old girl with a progressively increasing black mass in the upper nasal part above the cornea of the left eye. The biomicroscopy revealed an oval, bluish mass measuring 10x10x5 mm with congestion of the overlying conjunctiva. Conjunctival biopsy showed sporoblasts of *Rinosporidium seeberi*. Treatment was conducted by conjunctival resection and tectonic corneoscleral graft (13x13mm) over the staphyloma. Within 1 year of follow-up the patient presented a partial staphyloma reduction, 9x9x2.5 mm, and the patch detached from the lesion. A novel surgical approach was done reducing the corneal patch and no recurrence was seen after 9 months.

**Conclusions:**

This case is one of the largest anterior scleral staphylomas secondary to rhinosporidiosis described in the literature. Scleral anterior staphyloma partial regression is an unusual outcome after a tectonic corneoscleral graft. Infection resolution and graft covering of thinned area contributed to scleral reepithelization.

## Background

Rhinosporidiosis is a rare chronic infection of the mucous membrane that is caused by the *Rhinosporidium seeberi,* a microorganism of Mesomycetozoa clade. The common sites affected are nose and nasopharynx, although it may also be present on the eye, ear and even genitalia in both sexes. Approximately 15% of cases of rhinosporidiosis are ocular, and the tarsal conjunctiva is the most common site of infection followed by bulbar conjunctiva, lid, the lacrimal gland, and sclera. [1] The presumed mode of infection is from the aquatic habitat through traumatized epithelium. The disease is worldwide but endemic in Africa and Asia, and most frequent in south India and Sri Lanka. [2] However, sporadic occurrence of the ocular form has been reported in America, particularly United States and Canada. [3, 4]

Bulbar oculosporidiosis represents about 12.4% of the cases and usually presents as polypoidal and vascular masses that bleed even upon touch. The definitive diagnosis is made by microbiological scrapings and histopathological examination of resected tissues. Scleral melting associated with bulbar conjunctival rhinosporidiosis is rare, there have been only 11 cases of scleral melt and staphyloma formation reported in the literature. [1, 5–10] This report describes the partial regression of one of the largest scleral ectasia related to oculosporidiosis infection after a corneoscleral tectonic graft.

## Case presentation

**Fig. 1:**
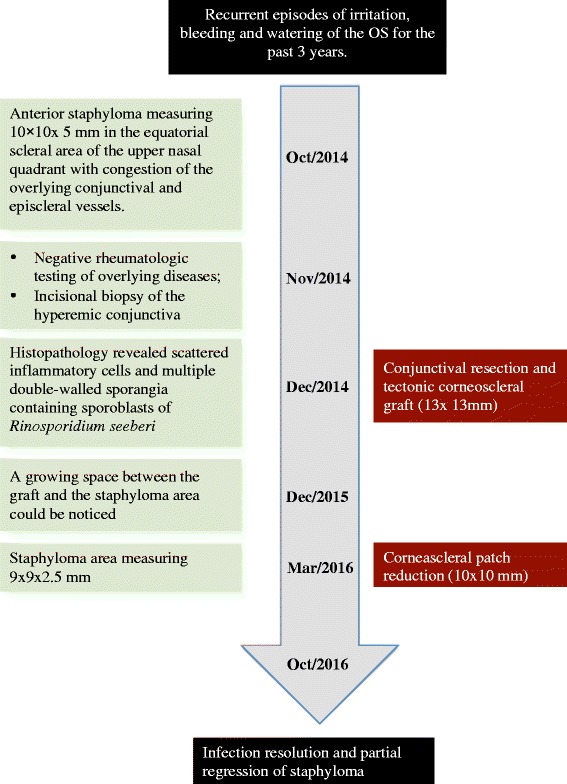
Timeline of diagnosis, interventions and outcomes

A 13-year-old girl from Presidente Juscelino, Maranhão, Brazil with recurrent episodes of irritation, bleeding and watering of the OS for the past 3 years (Fig. 1). She exhibited pain and a progressively increasing mass in the upper nasal area above her left cornea for 2 years. She often swims in ponds and rivers. There was no history of trauma, epistaxis, close contact with animals, or similar masses elsewhere in the body. No contributory medical illnesses were found during the investigation.

**Fig. 2:**
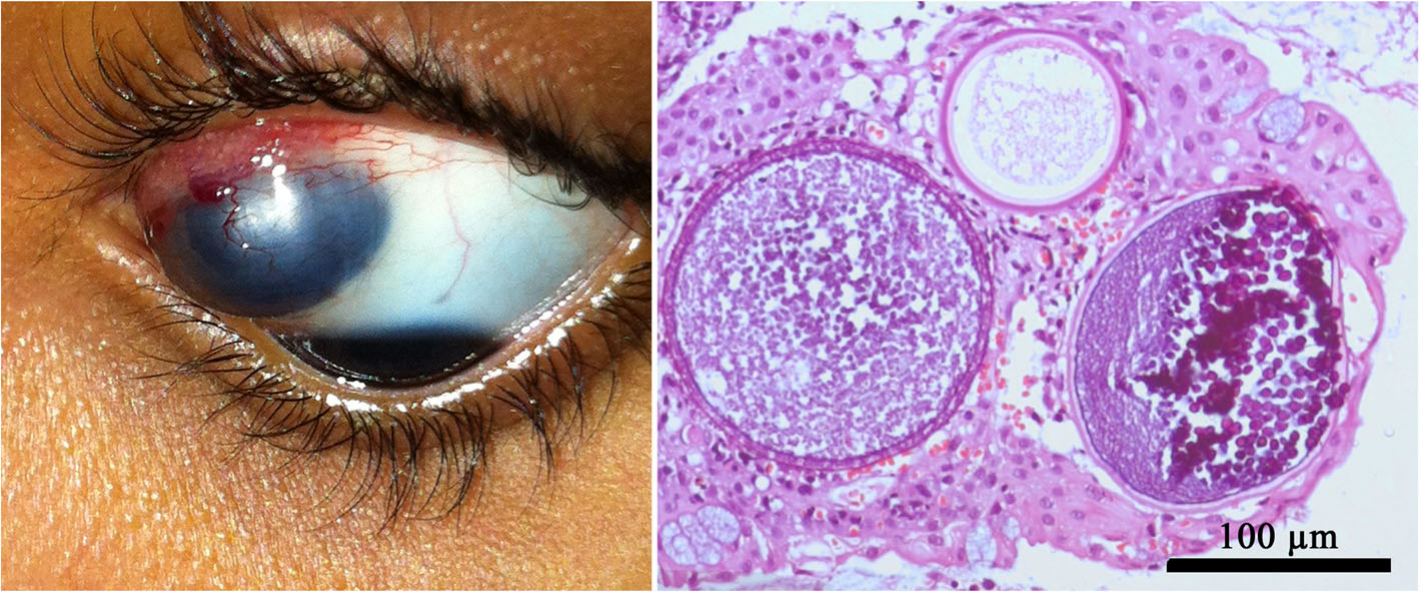
The anterior scleral staphyloma, 10x10x5mm, with an underlying hyperemic conjunctiva with scattered white corpuscular bodies (left). Histopathology (right) revealed sporangia of different sizes containing numerous endospores extends into the epithelium and are about to rupture and trans-epithelial elimination (haematoxylin–eosin; original magnification 400×)

On examination, uncorrected Snellen visual acuity was 20/20 in OD and the best-corrected visual acuity was 20/20 in OS with a − 4.75-diopter cylinder at 120 degrees. In OD, slit-lamp biomicroscopy, intraocular pressure and fundus examination were normal. In OS, an oval, bluish swelling measuring 10 × 10 mm and 5 mm in height was seen in the equatorial area of the upper nasal quadrant. There was congestion of the overlying conjunctival and episcleral vessels. Slit-lamp biomicroscopy revealed scattered white corpuscular bodies with less than 1 mm in diameter at the posterior margin of the hyperemic conjunctiva (Fig. 2). An anterior chamber reaction +/4+ was detected and the intraocular pressure was normal. Fundus examination revealed an upper nasal subretinal hypopigmentation. Evaluation by an ear, nose, and throat specialist revealed no lesions.

A complete rheumatologic evaluation of overlying diseases was performed with no positive results. Therefore, an incisional biopsy of the hyperemic conjunctiva was performed and revealed scattered inflammatory cells and multiple double-walled sporangia containing sporoblasts of *Rinosporidium seeberi* (Fig. 2). A conjunctival resection and a tectonic corneoscleral graft were advised to repair the staphyloma.

At surgery, the involved conjunctiva was excised with a 1 mm cauterized margin. Both superior and medial rectus muscles were preserved even though they were less than 1 mm from the ectasic area. A 13 × 13 mm corneoscleral tectonic graft was applied over the staphyloma wall and secured in place with interrupted 9–0 nylon sutures. Conjunctiva and tenon were mobilized to cover the scleral portion of the graft. Postoperatively, the patient was given topical antibiotic for 15 days and dexamethasone 0,1% eye drops for 2 months. No oral medications were prescribed. She was monitored weekly in the first month, then every 3 months until 1 year of the surgery.

**Fig. 3:**
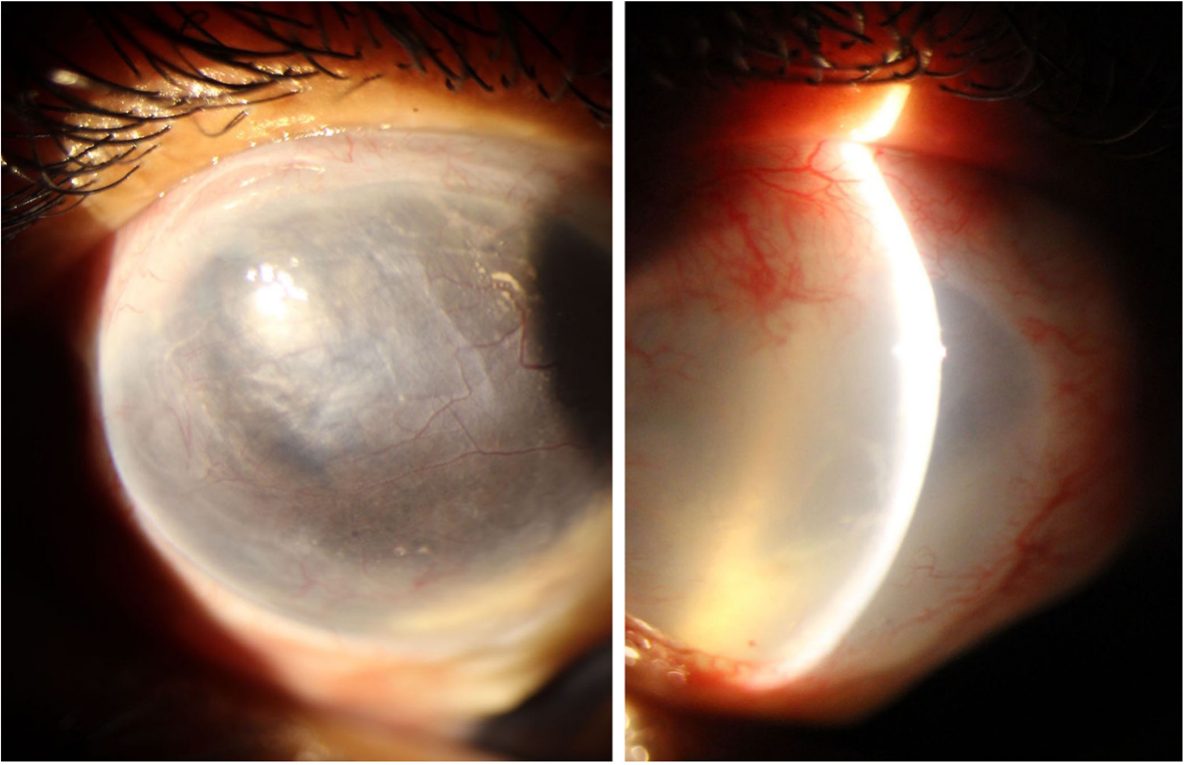
Sixth month postoperative corneoscleral tectonic aspect (left) no signs of rhinosporidiosis recurrence was seen. After 12 months (right) it was possible to observe a growing space between the graft and the staphyloma area

After 1 year, the staphyloma showed signs of reduction in height; a space was visualized between the lesion and the patch (Fig. 3). The cylinder decreased to − 2.50-diopter at 120 degrees. And another surgical approach was performed within 15 months to reduce the corneascleral patch. During the surgery was possible to separate the patch from the lesion; the staphyloma measured 9x9x2.5 mm and the graft was reduced to 10x10mm with continuous locking 9–0 nylon sutures. During the 9 months of follow-up, no signs of patch failure were found.

## Discussion

The *Rhinosporidium seeberi* thrives in a hot tropical climate. [11, 12] In Brazil, the state of Maranhão lies on the northeastern coast in the tropical zone. The temperature varies between 24 degrees and 33 degrees Celsius year-round. The majority of patients have previous history of bath in ponds and rivers; the conjunctival infection is explained by direct exposure of the conjunctiva to water during bathing. Although the majority of oculosporidioses cases are sporadic, the infection has higher prevalence in children and young adults between 3 and 39 years of age. [12]

The ocular structures are the second most commonly involved after the nasal mucosa. Although quite rare, when present, scleral ectasia is associated with scattered grey–white subepithelial conjunctival granules along the posterior margin of the defect. [12] Common differential diagnoses of scleral staphylomata in a quiet eye include scleromalacia perforans, hyaline plaques, and spontaneous intercalary perforation. No patients with oculosporidiosis and scleral involvement described in the literature had a history of associated rheumatoid arthritis.

The treatment of this condition is essentially surgical and during surgery it is important to completely excise the involved conjunctiva. The staphyloma repair of the scleral thinning over a large area did not allow a direct approximation of the edges. Therefore, a patch graft over the area was necessary. Castelino et al. [5] described three case reports of staphyloma related to rhinosporidiosis repaired with a scleral homograft; one had been previously treated with a periosteal graft and had a total recurrence of the ectasia. The greatest one (7 × 12 mm) had a symblepharon formation after 5-years. In all cases the conjunctiva was mobilized and sutured in place over the scleral grafts.

Covering the exposed sclera by either a conjunctival flap or a properly secured single/multilayered amniotic membrane is important to achieve a viable graft. There were at least two important challenges to tackle in this particular case. One was removing the tissue above the thinned area without perforate the eye, and the other was performing a conjunctival flap to cover a scleral graft because the conjunctiva over the lesion must be well resected to prevent recurrence. Jacob et al. [9] described another technique to cover the scleral staphyloma secondary to rhinosporidiosis. They performed a tectonic corneal graft over the ectasia. The advantage is that the cornea does not need to be covered, even with active inflammation. In the present case, the size of the lesion (10 × 10 × 5 mm) a corneoscleral graft of 13 × 13 mm was done to cover the ectasia especially because its height.

In the early postoperative period, the patient showed recovery without signs of infection recurrence or graft failure. Additionally, the corneal transparency of the patch was essential to observe the ectasia during follow-up. After 1 year, it was possible to visualize an enlarged space between the corneal graft and the staphyloma. During the second surgical intervention was possible to visualize a thicker sclera and a staphyloma height of 2,5 mm. This reduction was attributed to scleral fiber rearrangement, reepithelization and child eye growth.

## Conclusions

This case is one of the largest anterior scleral staphylomas described in the literature. Ocularsporidiosis scleral involvement was probably secondary to underlying chronic conjunctivitis. Treating large staphylomas is challenging, especially after infection. The use of corneascleral graft was crucial to observe the eventual recurrence of rhinosporidiosis and the partial regression of staphyloma.

## Abbreviations

OD: Oculus dexter (right eye)
OS: Oculus sinister (left eye).
